# First case of laboratory-confirmed severe fever with thrombocytopenia syndrome disease revealed the risk of SFTSV infection in Xinjiang, China

**DOI:** 10.1080/22221751.2019.1645573

**Published:** 2019-07-26

**Authors:** Liying Zhu, Feifei Yin, Abulimiti Moming, Jingyuan Zhang, Bo Wang, Lijuan Gao, Jianwen Ruan, Qiaoli Wu, Na Wu, Hualin Wang, Fei Deng, Gang Lu, Shu Shen

**Affiliations:** a State Key Laboratory of Virology and National Virus Resource Center, Wuhan Institute of Virology, Chinese Academy of Sciences, Wuhan, People’s Republic of China; b University of Chinese Academy of Sciences, Beijing, People’s Republic of China; c Hainan Medical University–The University of Hong Kong Joint Laboratory of Tropical Infectious Diseases, Hainan Medical University, Haikou, and the University of Hong Kong, Pokfulam, Hong Kong Special Administrative Region, People’s Republic of China; d Department of Pathogen Biology, Hainan Medical University, Haikou, People’s Republic of China; e Key Laboratory of Translational Tropical Medicine of Ministry of Education, Hainan Medical University, Haikou, People’s Republic of China; f Xinjiang Key Laboratory of Biological Resources and Genetic Engineering, College of Life Science and Technology, Xinjiang University, Urumqi, People’s Republic of China; g Department of Infectious Diseases, Haikou Hospital Affiliated to Xiangya School of Medicine, Central South University, Haikou, Peoples People’s Republic of China

**Keywords:** Severe fever with thrombocytopenia syndrome virus, first laboratory-confirmed case, Xinjiang, exposure to ticks, virus isolation

## Abstract

The Xinjiang Uygur Autonomous Region locating in Northwest of China was not considered the epidemic area of severe fever with thrombocytopenia syndrome (SFTS). Here we report the first laboratory-confirmed SFTS case that a female patient had tick bite in Xinjiang and illness onset after returning to Hainan Province. Laboratory tests identified SFTS virus (SFTSV) infection, and the virus was isolated from the patient’s serum sample. Furthermore, SFTSV prevalence among tick groups was identified, and IgM response to SFTSV from febrile patients was identified. The findings suggested that there have been risks of SFTSV infection due to exposure to ticks in Xinjiang.

Severe fever with thrombocytopenia syndrome (SFTS) is an acute infectious disease first reported in 2009, with clinical symptoms of high fever, thrombocytopenia, and leukocytopenia [1]. High fatality rates of 6–30% in China [1–3] and ∼30% in South Korea and Japan have been reported [4,5]. The SFTS virus (SFTSV) is the causative agent, which now is the representative species of genus *Banyangvirus* in family *Phenuiviridae* of order *Bunyavirales* according to the second update of the taxonomy of *Bunyavirales* in 2018 announced by International Committee on Taxonomy of Viruses (ICTV) [6]. Human infection of SFTSV could occur due to tick bites [1]. Until 2016, over 7000 SFTS cases were reported from 23 provinces in China. Laboratory-confirmed SFTS cases were recorded in 19 provinces in Central and Eastern China [3]. The Xinjiang Uygur Autonomous Region (Xinjiang) is not considered an epidemic area of SFTS. A few suspected cases with symptoms similar to SFTS were reported to the Chinese Disease Prevention and Control Information System, but molecular biological and virological evidence was lacking [3,7].

We report the first laboratory-confirmed SFTS case in Xinjiang. It was a 62-year-old woman who lived in Hainan Province and spent a 17-day holiday in Xinjiang in 2017. She and her husband flew from Haikou City in Hainan on 8 May. The trip started from Urumqi, went through Yuli, Bayanbulak Grassland, Korla, and Hejing, then passed by Urumqi to Kanas resort, and finished back to Urumqi (Figure 1(a,b)). On 23 May after returning to Urumqi, she recognized that her scalp was feeling itchy for >2 days. In that evening, two ticks were found in her hair before bathing and papules were noted on her scalp (Figure 1(c)). She visited the Traditional Chinese Medicine Hospital in Urumqi City where the papules were confirmed as traces of tick bites and were disinfected. In the evening of 25 May (day 1) when returning to Haikou City, she unexpectedly had fever (38.7°C), myalgia, and lumbago, and visited the Department of Infectious Diseases in Haikou hospital, where she was admitted due to fever of unknown origin. Lyme disease, tick-borne relapsing fever, and scrub typhus were suspected because of the history of tick bite but were not supported by clinical examinations. Cytomegalovirus, Epstein–Barr virus, and bacterial infections were excluded by ELISA and bacteria culture with venous blood. A routine blood test showed leukopoenia, thrombocytopenia, and lymphocytopenia (Supplementary Table S1). She had rheumatoid arthritis as revealed by increasing levels of C-reactive protein and complement C1q. She had stabilized on the use of disease-modifying antirheumatic drugs (methotrexate and leflunomide) and achieved rheumatoid arthritis remission. She was administered paracetamol and caffeine tablets, doxycycline hydrochloride, and supportive treatment. She recovered and was discharged on 7 June. Figure 1.(a) A map presenting the patient’s travelling routes in Xinjiang and (b) the timeline of important events before illness onset and during hospitalization. The green star represents the grasslands and the green triangle indicates the location of poplar tree forest. KNS, Kanas resort; URQ, Urumqi City; HJ, Hejing County; KL, Korla City; BKG, Bayanbulak Grassland; YL, Yuli County. XJ, Xinjiang Uygur Autonomous Region; HN, Hainan Province. (c) The images of one tick found from the patient’s hair (left) and the papules noted on her scalp (right) were taken by using a mobile phone (HUAWEI EVA-AL10). (d) SFTSV particles observed by negative staining electron microscopy. Bar, 200 nm. (e) The maximum likelihood phylogenetic trees were created based on the complete sequences of the L (left), M (middle) and S (right) segments, respectively. The branches of genotypes are indicated by colours. The isolated virus strain XJ/HN2017 is labelled with a red solid circle. Trees were constructed using Mega 5.0 and tested by bootstrap method of 1000 replicates. Bootstrap values >50% are shown at each node.

**Figure:**
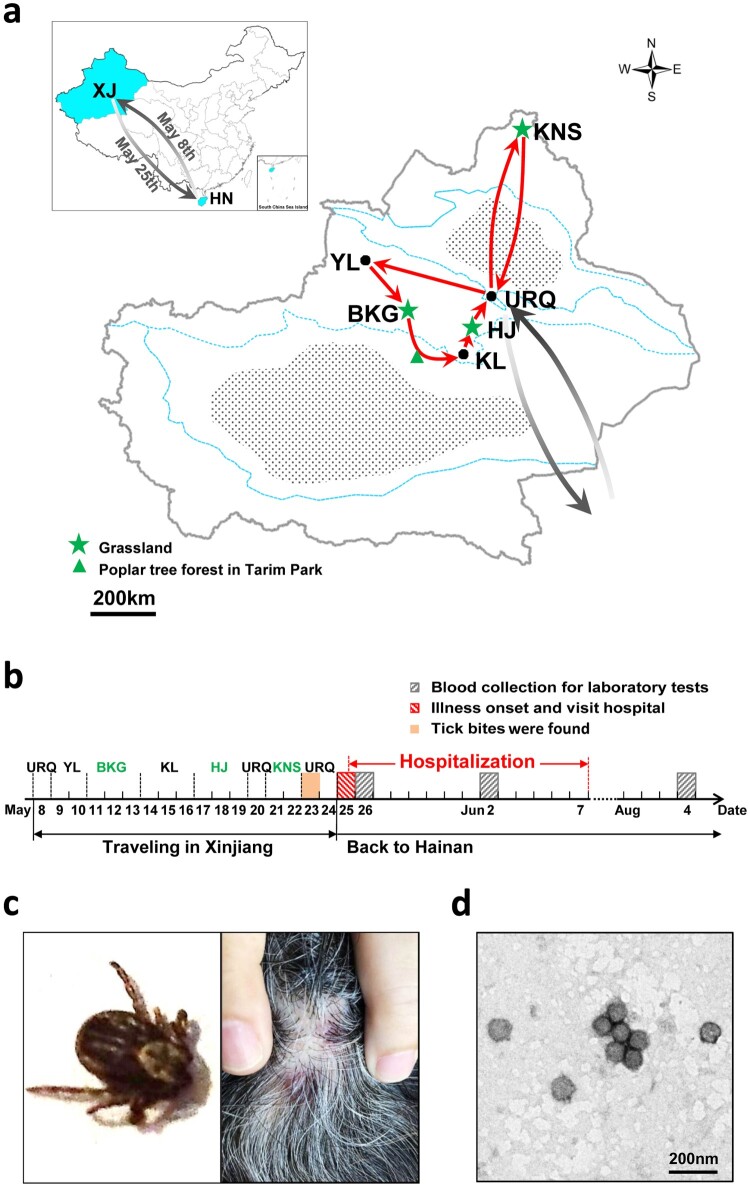

**Figure:**
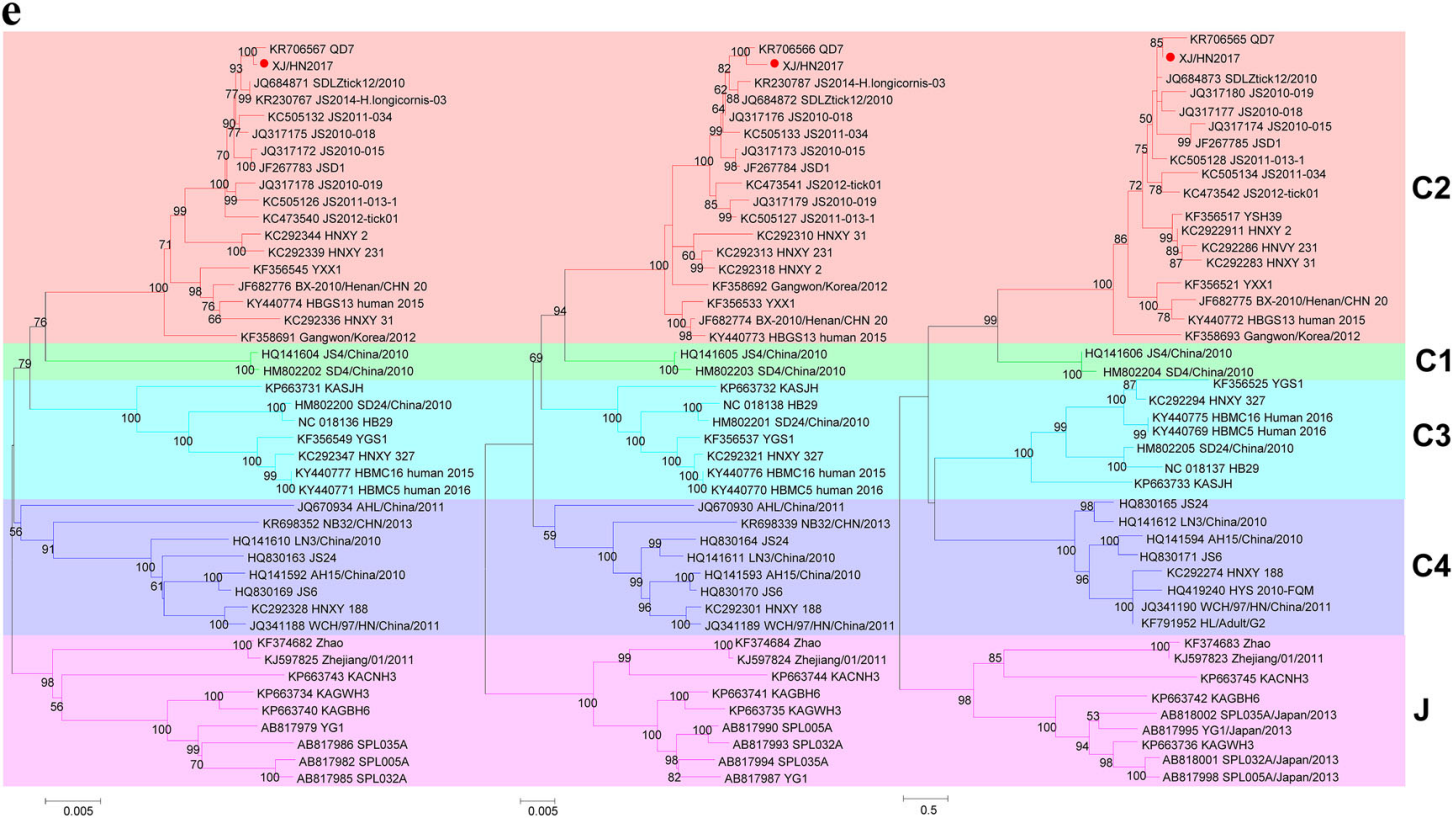

Laboratory tests were performed using blood samples collected on day 2 (26 May) and day 9 (2 June) after illness onset and on day 72 (8 August) during a follow-up visit (Supplementary Table S2). Infection of *Anaplasma* spp. and *Ehrlichia* spp., Crimean-Congo haemorrhagic fever virus, Guertu virus, Dengue virus, and Influenza A virus was excluded (data not shown). The SFTSV RNA virus load was 7.55 ± 2.54 × 10^6^ and 4.27 ± 1.35 × 10^6^ copies/mL on days 2 and 9, respectively. Both serum samples were anti-SFTSV IgM positive with neutralization activity titres of 2^4^ and 2^5^ on days 2 and 9, respectively; however, anti-SFTSV IgG was undetectable at the current dilutions (1:100). SFTSV RNA and antibodies were undetectable from the last sample (day 72). Further, SFTSV was isolated from the sample collected on day 2. Increasing cell infection and SFTSV RNA copies in culture supernatants were detected from each passage by immunofluorescence assay and quantitative reverse transcription-PCR, respectively (data not shown). Virions were purified from culture supernatants and visualized having typical bunyavirus morphology by negative staining electron microscope (Figure 1(d)). The virus genome contains S, M, and L segments in the length of 1745, 3378, and 6368 nt, respectively. Blast comparison showed that the new strain (XJ/HN2017) is close to SFTSV from Shandong and Jiangsu. The S segment shared high nucleotide similarity to strain SDLZtick12/2010 derived from ticks (99.77%), while M and L segments were highly similar to strain QD7 from a patient (99.53% and 99.75%, respectively). It belongs to C2 genotype based on the maximum likelihood trees of S, M, and L segments (Figure 1(e)). These results suggested that the patient was infected with SFTSV.

This is the first laboratory-confirmed SFTS case in Xinjiang presenting with mild symptoms. The patient suffered from rheumatoid arthritis, which is a chronic, progressive, and systemic autoimmune disease resulting from T-regulatory and B-regulatory cell dysfunction [8,9]. A recent study reported that the impairment of T cell subsets would impair B cell immunity and the antibody response to SFTSV [10], which indicated that levels of IgG response from this case might be extremely low so that was undetected. Otherwise, individual differences and virus strains may be related to clinical manifestations of SFTS. The patient might be infected due to tick bite in Xinjiang. Unfortunately, the two ticks found in her hair were not collected for further tests. The tick species could not be identified on the basis of morphology because of the limited resolution of the image taken by the mobile phone (HUAWEI EVA-AL10) (Figure 1(c), left). Nevertheless, subsequent investigations found the SFTSV prevalence among tick groups collected from Xinjiang in 2017 (Supplementary). Of the 259 tick groups, 91 groups were SFTSV positive, including 20 groups of *Dermacentor nuttalli* and 71 groups of *Hyalomma asiaticum* (Supplementary Table S3). The actual minimum infection rate, which assumes that an SFTSV-positive group contained 1 infected tick [11], may be 100 times lower because the tests were not performed on individuals but among groups (*n* = ∼100 per group). From the PCR products of 11 randomly selected tick groups (6 groups of *D. nuttalli* and 5 groups of *Hy. asiaticum*), the consensus sequence of both partial S and L segments (XJ/Tick-05 and XJ/Tick-01) was obtained, which shared 92.96% and 95.85% identity to strain XJ/HN2017, respectively; meanwhile, three consensus partial M sequences (XJ/Tick-02, XJ/Tick-198, and XJ/Tick-69) were obtained, showing 96.29%, 96.13%, and 96.13% identities to strain XJ/HN2017 (Table S4). *D. nuttalli* and *Hy. asiaticum* are the dominant tick species in Xinjiang. *D. nuttalli* is often found in grasslands, while *Hy. asiaticum* is active in poplar tree (*Populus euphratica*) forests of desert and semi-desert areas [12]. The patient visited grasslands in Bayanbulak Grassland (11–13 May), Hejing County (17–19 May), and Kanas Resort (21–22 May) and had a short stay (1–2 h) in poplar tree forest of Tarim Park on the way from Bayanbulak Grassland to Korla City (Figure 1(a,b)). She might be exposed to ticks 3–14 days before illness onset. Moreover, serological investigation found four of the 87 archived serum samples collected from febrile patients in 2007 were tested IgM positive (4.60%), including 3 women, aged 24–35 years, and 1 patient with unknown personal data (Supplementary Table S5). IgG was not detected from all patients, neither was neutralization activity from the four IgM positive samples. Cross-reaction of the four IgM positive samples to Guertu virus was excluded, a novel virus found in Xinjiang closely related to SFTSV (data not shown) [13]. These samples have been preserved for more than a decade and might have undetectable reaction to virus antigen. Our findings, for the first time, revealed the SFTSV prevalence among ticks and the potentials of human infection with SFTSV ever since 2007 in Xinjiang.

In summary, we reported the first laboratory-confirmed SFTS case and epidemiological data of SFTSV in Xinjiang, China, which revealed the distribution of SFTSV in northwestern China and substantial risks of SFTSV infection to humans. Therefore, the public, clinical doctors, disease control specialists, and researcher could take appropriate strategies to avoid tick exposure, carry out disease diagnosis and treatment, prevent disease outbreak and epidemics, and perform further surveys in northwestern areas of China.

## Supplementary Material

Supplemental Material
