# Supplementary material for: First case of laboratory-confirmed severe fever with thrombocytopenia syndrome disease revealed the risk of SFTSV infection in Xinjiang, China
Source: Emerg Microbes Infect. 2019 Jul 26;8(1):1122–5. doi: 10.1080/22221751.2019.1645573 (PMC6711166; doi:10.1080/22221751.2019.1645573)
Supplement: Supplemental Material [file TEMI_A_1645573_SM8403.docx]

**Supplementary**

**Materials and Methods**

**Laboratory tests with the patient’s serum samples**

The blood samples were separated into blood cells and serum by centrifugation after incubation at 37°C for 1 hour. DNA was extracted from blood cells using Viral RNA/DNA Kits (Invitrogen, Carlsbad, CA, USA) according to the manufacturer’s instruction. Potential rickettsia infection was examined using DNA templates and universal primers by PCR according to the technical guideline for the prevention and control of human granulocytic anaplasmosis (HGA), which was issued by the state health and family planning commission of China in 2008 [1]. For detection of RNA viruses, RNA was purified from 50 μL serum using TRIzol (Invitrogen) according to the manufacturer’s instruction. Potential infection of RNA viruses, including Crimean-Congo Hemorrhagic fever virus, Guertu virus, severe fever with thrombocytopenia syndrome virus (SFTSV; S, M, and L segments, respectively), Dengue virus, and Influenza A virus, was tested by nested reverse transcription (RT)-PCR and primers described in Supplementary Table S6 [1-6]. SFTSV RNA in the patient serum was further determined by quantitative RT-PCR (qRT-PCR). Total RNAs were purified from 100 μL samples using the PureLink™ RNA Mini Kit (Thermo Fisher Scientific, Waltham, USA). The plasmid pT-Easy-SFTSV S^809-1350^ with the insertion of a fragment of partial S segment (809–1350 nt) was constructed and used as the DNA template to generate standard RNA by *in vitro* transcription assay using a Transcript Aid T7 high yield transcription kit (Thermo Fisher Scientific) according to the manufacturer’s instruction. SFTSV RNA copies were determined by qRT-PCR in a volume of 20 μL mixture with primers: SFTSV-F: 5′-TTCACAGCAGCATGGAGAGG-3′ and SFTSV-R: 5′-GATGCCTTCACCAAGACTATCAATG-3′ using the HiScript II One Step qRT-PCR SYBR Green Kit (Vazyme Biotech, Nanjing, China) according to the manufacturer’s instruction. Serological examinations were performed to detect IgM and IgG response to SFTSV with the serum samples at 1:100 dilution using both immunofluorescence assay as previously described [7] and ELISA methods using the commercial human anti-SFTSV IgG or IgM ELISA kits (NewZongke, Wuhan, China). Microneutralization assay was performed as described [8].

**Virus isolation and characterization**

Serum samples were used for virus isolation as described [6]. To identify virus isolation, a small portion of cells from each passage were subcultured in a 35-mm dish, from which SFTSV infection was examined by immunofluorescence assay. The increase of SFTSV RNA in supernatants from each passage was detected by qRT-PCR. Virus particles were purified and visualized by negative staining electron microscope as described [8]. Complete sequence of the isolated SFTSV strain was sequenced as described [8]. Phylogenetic analyses were conducted with full-length sequences of SFTSV S, M and L segments as described [9].

**Detection of SFTSV RNA in tick groups from XJUAR**

A total of 25,578 ticks were collected from shrubs in the wild field in Xinjiang during April and May, 2017, when they were questing for a blood meal. Tick species were identified under a microscope according to morphology by an experienced technician and were confirmed by PCR using primers TITS2 F1 and TITS2 R1 to amplify a partial fragment of the second internal transcribed spacer (ITS2) regions as described [10]. These ticks were grouped into 259 groups according to species and sampling locations (unpublished data). Homogenates from each group were preserved by the National virus resource center, Wuhan Institute of Virology, Chinese Academy of Science. Two tick species were involved in this study, including 163 groups of 19,500 *D. nuttalli* ticks and 96 groups of 2,988 *Hy. asiaticum* ticks (Supplementary Table S3).

Total RNA was purified from 100 μL homogenates from each group using TRIzol (Invitrogen) according to the manufacturer’s instructions. One microgram of RNA was subjected to cDNA synthesis with random primers using M-MLV (Invitrogen) according to the manufacturer’s instructions. Nested-PCR was then performed to detect the SFTSV L segment with primers listed in Supplementary Table S6 using the 2 × Rapid Taq Master Mix (Vazyme Biotech) according to the manufacturer’s instructions. Eleven SFTSV L RNA positive groups were randomly selected to detect S and M segments using primers listed in Table S6. PCR products of the partial S (positions 1251-1449nt), M (positions 2482-3101nt), and L (positions 589-806nt) segments were sequenced and aligned. The consensus sequences were used for further sequence analysis after the primer sequences were wiped off.

**Serological examination with patients’ serum samples from XJUAR**

A total of archived 87 serum samples from patients with fever of unknown origin were collected from clinics in Bachu County locating in Junggar Basin, XJUAR in June 2007 and were preserved at -80 °C until further examination. Unfortunately, 38 samples had missing data regarding gender and age. Information regarding the remaining samples is summarized (Supplementary Table S4). Immunofluorescence assay was performed to detect Anti-SFTSV IgG and IgM as previously described [7]. The IgM positive samples were further tested by microneutralization assay as described [8].

**Bio-informatic analyses of viral sequences**

Sequence comparison was performed using Blastn Comparison (NCBI). Sequence alignments were performed using ClustalW. Maximum-likelihood (ML) trees were constructed using Mega 6.0 and were tested by bootstrapping with 1000 replicates. Sequences of the new isolate (XJ/HN2017) (MK300945 to MK300947) and the consensus partial sequences (MN078155 to MN078159) obtained from ticks were deposited in GenBank.

**Ethical statement**

Studies using human serum samples were reviewed and approved by the ethics committees of Wuhan Institute of Virology, Chinese Academy of Sciences (Approval number: WIVH01201501). Written informed content was provided by adult participants and parents on behalf of child participants under 18 years old.

**References**

1. Nhc.gov.cn [Internet]. Beijing: General Office of the Ministry of Health of the People's Republic of China. Technical Guidelines for the Prevention and Control of Human Granulocytic Anthroposis (Trial) (2008 edition). [updated 2008 Feb 26; cited 2019 Mar 18]. Available from: http://www.nhc.gov.cn/yjb/s3577/200804/c419dbb1a2a8447d85f63e483719bf98.shtml.

2. Chinacdc.cn [Internet]. Beijing: China Center for Disease Control and Prevention. National Dengue Fever Surveillance Programme (Trial). [cited 2019 Mar 18]. Available from:

http://www.chinacdc.cn/jkzt/crb/gjfd/zl/dgr/jc/200508/t20050810_24741.html.

3. Chinacdc.cn [Internet]. Beijing: China Center for Disease Control and Prevention. Technical Guidelines for National Influenza Surveillance (2017 edition) [cited 2019 Mar 18]. Available from:

<http://ivdc.chinacdc.cn/cnic/zyzx/jcfa/201709/t20170930_153976.htm>.

4. Shen S, Duan XM, Wang B, et al. A novel tick-borne phlebovirus, closely related to severe fever with thrombocytopenia syndrome virus and Heartland virus, is a potential pathogen. Emerg Microbes Infect. 2018; 7: 95-108.

5. Guo R, Shen S, Zhang YF, et al. A new strain of Crimean-Congo hemorrhagic fever virus isolated from Xinjiang, China. Virol Sin. 2017; 32: 80-88.

6. Zhang YF, Shen S, Shi JM, et al. Isolation, characterization, and phylogenic analysis of three new severe fever with thrombocytopenia syndrome bunyavirus strains derived from Hubei Province, China. Virol Sin. 2017; 32: 89-96.

7. Huang DY, Jiang YP, Liu XP, et al. A Cluster of Symptomatic and Asymptomatic Infections of Severe Fever with Thrombocytopenia Syndrome Caused by Person-to-Person Transmission. Am J Trop Med Hyg. 2017; 97: 396-402.

8. Yu XJ, Liang MF, Zhang SY, et al. Fever with thrombocytopenia associated with a novel bunyavirus in China. N Engl J Med. 2011; 364:1523-1532.

9. Shi JM, Hu S, Liu XP, et al. Migration, recombination, and reassortment are involved in the evolution of severe fever with thrombocytopenia syndrome bunyavirus. Infect Genet Evol. 2017; 47:109-117.

10. Lu X, Lin XD, Wang JB, et al. Molecular survey of hard ticks in endemic areas of tick-borne diseases in China. Ticks Tick Borne Dis. 2013; 4(4):288-296.

**Supplementary Table S1. Clinical parameters of the patient with severe fever with thrombocytopenia syndrome**

| **Clinical parameters*** | **Day 1**  **(May 25)** | **Day 2**  **(May 26)** | **Day 4**  **(May 28)** | **Day 12**  **(Jun 5)** | **Normal range** |
| --- | --- | --- | --- | --- | --- |
| White blood cells (×10^9^ /L) | 5.07 | NA | 2.66↓ | 7.44 | 3.5–9.5 |
| Platelets (×10^9^/L) | 151 | NA | 94↓ | 298 | 125–350 |
| Lymphocytes (×10^9^/L) | 0.94↓ | NA | 0.41↓ | 2.01 | 1.1–3.2 |
| Monocytes (×10^9^/L) | 0.40 | NA | 0.24 | 0.60 | 0.1–0.6 |
| Neutrophils (×10^9^/L) | 3.70 | NA | 1.99 | 4.68 | 1.8–6.3 |
| CRP (mg/L) | 20↑ | NA | NA | 10 | 0–10 |
| C1q (mg/L) | 268.94↑ | NA | 310.62↑ | NA | 0–10 |
| ALT (U/L) | NA | 27 | NA | 16 | 7–45 |
| AST (U/L) | 22 | 23 | NA | 21 | 13–40 |
| CK (U/L) | 55 | NA | NA | 46 | <190 |
| CK-MB (U/L) | 9.0 | NA | NA | 9 | 0–25 |
| Creatinine (mg/L) | 70 | NA | 66 | 72 | 44–133 |
| BUN (mmol/L) | 2.80 | NA | 0.7↓ | 1.7↓ | 1.78–6.8 |

Levels of clinical parameters higher than the normal range are indicated by up arrow, while those decreased below the normal are indicated by down arrow. CRP, C-reactive protein; C1q, complement; ALT, alanine aminotransferase; AST, aspartate aminotransferase; CK, creatine kinase; CK-MB, creatine kinase MB; BUN, blood urea nitrogen; NA, not available.

**Supplementary Table S2. Laboratory tests using serum samples from the patient**

| **Laboratory tests** | | **Day 2**  **(May 26)** | **Day 9**  **(Jun 2)** | **Day 72**  **(Aug 4)** |
| --- | --- | --- | --- | --- |
| **PCR detection of possible pathogens** | | | | |
| SFTSV | L segment | + | - | - |
|  | M segment | - | + | - |
|  | S segment | + | - | - |
| **Quantitative reverse transcription-PCR of SFTSV RNA** | | | | |
| Virus loads (copies/mL) | | 7.55±2.54×10^6^ | 4.27±1.35×10^6^ | N/A |
| IgM | ELISA | 2^6^ | 2^6^ | - |
|  | IFA | + | - | - |
| IgG | ELISA | - | - | - |
|  | IFA | - | - | - |
| **Neutralization** | | 2^4^ | 2^5^ | N/A |

ELISA, enzyme-linked immuno sorbent assay; IFA, immunofluorescence assay. N/A, not applicable.

**Supplementary Table S3. Epidemiological Investigation of severe fever with thrombocytopenia syndrome virus in Xinjiang Uygur Autonomous Region, China, 2017**

| **Tick species** | **Tick numbers** | **Groups** | **SFTSV positive groups (Minimum infection rate, %)*** |
| --- | --- | --- | --- |
| *Hy. asiaticum* | 2988 | 96 | 71 (27.41%) |
| *D. nuttalli* | 19500 | 163 | 20 (7.72%) |
| Total | 25578 | 259 | 91 (35.14%) |

*Positive percent based on RT-PCR detection for SFTSV L segment.

The minimum infection rate was expressed by the number of positive groups over total groups.

**Supplementary Table S4. Comparison of consensus sequences of the partial S, M, and L segments from RT-PCR products with strain XJ/HN2017**

| **Segment** | **Consensus sequence** | **Tick species^#^** | **Nucleotide positions (length)** | **Identity to strain XJ/HN2017** |
| --- | --- | --- | --- | --- |
| L | XJ/Tick-01 | Both | 589-806nt (218nt) | 95.85% |
| M | XJ/Tick-02 | Both | 2482-3101nt (620nt) | 96.29% |
|  | XJ/Tick-198 | *Hy. asiaticum* |  | 96.13% |
|  | XJ/Tick-69 | *D. nuttalli* |  | 96.13% |
| S | XJ/Tick-05 S | Both | 1251-1449nt (199nt) | 92.96% |

^#^It presented tick species of the groups that the consensus sequences were obtained from. “Both” means that the consensus sequence were from both *Hy. asiatucum* and *D. nuttalli*.

**Supplementary Table S5. Results from human serum sample analyses from Xinjiang Uygur Autonomous Region**

| **Gender** | **Age (years)** | **Serum samples** | **Positive antibodies against SFTSV** | |
| --- | --- | --- | --- | --- |
|  |  |  | IgG (%) | IgM (%) |
| Male | 6–71 | 20 | 0 | 1 (5.00%) |
| Female | 1–75 | 29 | 0 | 1 (3.45%) |
| Unrecorded | N/A | 38 | 0 | 1 (2.63%) |
| **Total** | 1–83 | 87 | 0 | 3 (3.45%) |

N/A, not available.

**Supplementary Table S6. Sequences of primers to detect potential pathogens used in this study**

| **Virus** | | **Primers** | **Sequence (5’-3’)*** | **Length of product (bp)** |
| --- | --- | --- | --- | --- |
| *Anaplasma* spp. / *Ehrlichia* spp. | | Eh-out1  Eh-out2 | TTGAGAGTTTGATCCTGGCTCAGAACG  CACCTCTACACTAGGAATTCCGCTATC | 653 |
|  |  | Eh-gs1  Eh-gs2 | GTAATACTGTATAATCCCTG  GTACCGTCATTATCTTCCCTA | 282 |
|  |  | HGA1  HGA2 | GTCGAACGGATTATTCTTTATAGCTTG  TATAGGTACCGTCATTATCTTCCCTAC | 389 |
| Dengue virus | | +)  -) | GTGCACACATTGACAGAACA  CTTTCTATCCAATAACCCAT | 539 |
| Influenza A virus | | FluA-Forward  FluA-Reverse | GACCRATCCTGTCACCTCTGAC  GGGCATTYTGGACAAAKCGTCTACG | 105 |
| Guertu Virus | | C9F1  C9R1 | TTGCCATAGGGAATATGCTTC  TGGGAGGTGATGATTGGATG | 389 |
|  |  | C9F2  C9R2 | GGAGGTGATGATTGGATGA  TTCTGGTGGGAAATTAGACAC | 294 |
| CCHFV | | CCHFV-S-F2  CCHFV-S-R3 | TGGACACCTTCACAAACTC  GACAAATTCCCTGCACCA | 530 |
|  |  | CCHFV-S-F3  CCHFV-S-R2 | GAATGTGCATGGGTTAGCTC  GACATCACAATTTCACCAGG | 226 |
| SFTSV | L segment | SFTSV-L-F  SFTSV-L-R | TTGGCGTWATTGTAGTCTCGTC  AATGATGTTTGCTTGTTGCTCCT | 476 |
|  |  | SFTSV-L-nest-F  SFTSV-L-nest-R | CAGGATGAGGCAGAGGAGCT  TTGAGGGTCTTGGTGATGAAGT | 260 |
|  | M segment | SFTSV-M-F  SFTSV-M-R | AGCGAGGARAGTGCCCGTAC  AAGCCAGCTTYGTCCTYGATC | 791 |
|  |  | SFTSV-M-nest-F  SFTSV-M-nest-R | CGTACCATAAAAAAAGATGGGTG  CTCAGCCCATTGCCAAACAAG | 656 |
|  | S segment | SFTSV-S-F  SFTSV-S-R | GGCTCCRCGCATCTTCACATT  CATCATTGTCTTTGCCCTGACT | 397 |
|  |  | SFTSV-S-nest-F  SFTSV-S-nest-R | CCCCYGCAGTTGGAATTAGG  CAGGGTCHAAGAGGTTGATGG | 240 |

*The primers were used for detection of potential pathogens according to previous studies and the national technical guidelines [1-6].

CCHFV, Crimean-Congo Hemorrhagic fever virus; SFTSV, severe fever with thrombocytopenia syndrome virus.
